# Organization of the mitochondrial genomes of whiteflies, aphids, and psyllids (Hemiptera, Sternorrhyncha)

**DOI:** 10.1186/1471-2148-4-25

**Published:** 2004-08-03

**Authors:** MyLo L Thao, Linda Baumann, Paul Baumann

**Affiliations:** 1 Microbiology Section, University of California, One Shields Ave., Davis, California, USA, 95616-8665

## Abstract

**Background:**

With some exceptions, mitochondria within the class Insecta have the same gene content, and generally, a similar gene order allowing the proposal of an ancestral gene order. The principal exceptions are several orders within the Hemipteroid assemblage including the order Thysanoptera, a sister group of the order Hemiptera. Within the Hemiptera, there are available a number of completely sequenced mitochondrial genomes that have a gene order similar to that of the proposed ancestor. None, however, are available from the suborder Sternorryncha that includes whiteflies, psyllids and aphids.

**Results:**

We have determined the complete nucleotide sequence of the mitochondrial genomes of six species of whiteflies, one psyllid and one aphid. Two species of whiteflies, one psyllid and one aphid have mitochondrial genomes with a gene order very similar to that of the proposed insect ancestor. The remaining four species of whiteflies had variations in the gene order. In all cases, there was the excision of a DNA fragment encoding for cytochrome oxidase subunit III(*COIII*)-tRNA^gly^-NADH dehydrogenase subunit 3(*ND3*)-tRNA^ala^-tRNA^arg^-tRNA^asn ^from the ancestral position between genes for ATP synthase subunit 6 and NADH dehydrogenase subunit 5. Based on the position in which all or part of this fragment was inserted, the mitochondria could be subdivided into four different gene arrangement types. PCR amplification spanning from *COIII *to genes outside the inserted region and sequence determination of the resulting fragments, indicated that different whitefly species could be placed into one of these arrangement types. A phylogenetic analysis of 19 whitefly species based on genes for mitochondrial cytochrome b, NADH dehydrogenase subunit 1, and 16S ribosomal DNA as well as cospeciating endosymbiont 16S and 23S ribosomal DNA indicated a clustering of species that corresponded to the gene arrangement types.

**Conclusions:**

In whiteflies, the region of the mitochondrial genome consisting of genes encoding for *COIII-tRNA*^*gly*^-*ND3-tRNA*^*ala*^-*tRNA*^*arg*^-*tRNA*^*asn *^can be transposed from its ancestral position to four different locations on the mitochondrial genome. Related species within clusters established by phylogenetic analysis of host and endosymbiont genes have the same mitochondrial gene arrangement indicating a transposition in the ancestor of these clusters.

## Background

Whiteflies, psyllids, and aphids correspond to superfamilies within the suborder Sternorrhyncha (Hemiptera) [1]. These insects share a number of common properties that are a consequence of their utilization of plant phloem as their diet. This mode of feeding is accomplished by means of needle-like stylets that probe plant tissues between plant cells until they enter the phloem-sieve elements. Due to this mode of feeding, some species are of major agricultural importance in that they vector plant pathogens and in high numbers may cause plant debilitation due to excessive nutrient consumption [1]. Whiteflies, psyllids, and aphids have an obligatory association with prokaryotic endosymbionts localized in specialized cells called bacteriocytes that constitute a larger structure called the bacteriome [2-4]. In the past, numerous studies have been performed on the phylogeny of some of these endosymbionts and their hosts [2-5]. The results have indicated congruence between the endosymbiont and the host derived phylogeny. This observation has been interpreted as being the consequence of an infection of an insect ancestor by a prokaryote and the vertical transmission of the endosymbiont resulting in cospeciation or cocladogenesis. In a recent study of whiteflies, we compared the phylogeny based on endosymbiont 16S*-23S* rDNA to the phylogeny of the host based on several mitochondrial genes [6]. During this study, we found that in the whitefly, *Bemisia tabaci*, the order of some of the mitochondrial genes was quite different from the frequently found order of genes in the mitochondria of the class Insecta. This observation led us to obtain the full sequence of the mitochondrial genome of representatives of the suborder Sternorrhyncha. Due to the observed differences in the order of genes in the mitochondrial genome of whiteflies, we obtained additional mitochondrial sequences from species representative of the major phylogenetic clusters previously established on the basis of whitefly mitochondrial and endosymbiont genes [6]. Previous studies of the phylogenetic relationships of member of the Sternorrhyncha, using host 18S rDNA, indicated that it is a monophyletic group [7-9]. These studies also showed that aphids and whiteflies were more closely related to each other than to psyllids.

In animals, the mitochondrial genome is generally circular (14–17 kb), is maternally transmitted and has a relatively simple genetic structure, and a rapid rate of sequence change [10-12]. Of the thirty seven genes found in animal mitochondria, thirteen encode for proteins, consisting of three subunits of cytochrome oxidase (*COI*, *COII*, *COIII*), two subunits of ATP synthase (*atp6*, *atp8*), seven subunits of NADH dehydrogenase (*ND1*, *ND2*, *ND3*, *ND4*, *ND4L*, *ND5*, *ND6*), and cytochrome b (*cytB*). Two genes encode for the large subunit of ribosomal RNA (*16S*) and the small subunit of ribosomal RNA (*12S*). In addition, there are 22 tRNAs, two for leucine and two for serine, and one tRNA each for the remaining eighteen amino acids. In general, there is conservation of the gene order within phyla but variation between phyla [10,13-17]; the tRNA genes are subject to more change in their position than the genes for proteins and rRNAs. The order of mitochondrial genes has been suggested to be a good phylogenetic marker for studies of relationships [14]. The animal mitochondrial genome is generally very compact with few if any intergenic spaces. Usually there is one (or rarely more) noncoding region, frequently following *12S rDNA*. Such a region most often has a reduced G+C content and all or some of the following properties: a) direct repeats, b) inverted repeats, c) stretches of "T"s, "A"s, or "TA"s. By analogy with other well-studied mitochondria, such a region is considered to be a putative origin of DNA replication and a region from which transcription is initiated [10,12]. Variation in mitochondrial size is generally a consequence of variation in the length of the repeats in the noncoding region and not in the number of structural genes. Early studies within the class Insecta suggested conservation of the gene order over a wide range of different organisms indicating an ancestral gene order for this group [10,18]. However, more recent studies have shown that within the Hemipteroid assemblage, there is considerable variation in the order of genes in the orders Phthiraptera, Psocoptera, and Thysanoptera, but no variation in the order Hemiptera (that includes the suborder Sternorrhyncha) [18-21]. The complete sequence of the mitochondria of a representative of the Phthiraptera (wallaby louse) and the Thysanoptera (plague thrips) has been obtained [18,20]. The latter shows major differences from the ancestral gene order. The Hemiptera and the Thysanoptera are sister groups and it was consequently of interest to obtain sequences of the mitochondrial genomes of the former. Since the sequence of mitochondrial genomes is poorly conserved, sequence determination of a portion of the genome is useful for the study of closely related species or the population structure within a species [22,23]. The availability of completely sequenced mitochondrial genomes is also an aid to the design of primers for the PCR amplification of the regions selected for population studies.

## Results

### Evolutionary relationships within the Sternorrhyncha

**Figure 1:**
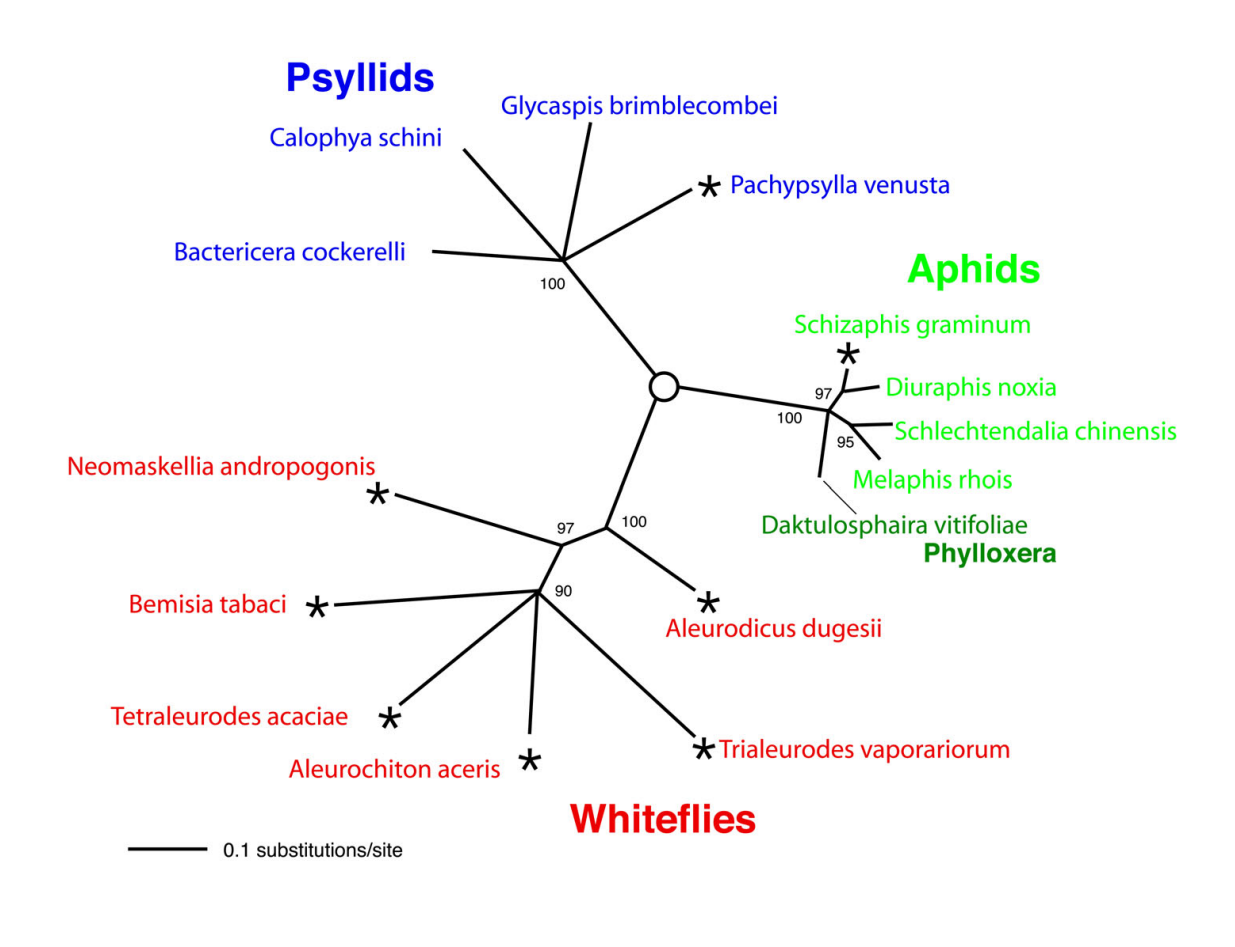
**Unrooted phylogenetic tree showing the relationships of members of the Sternorrhyncha (whiteflies, aphids, and psyllids)**. The tree is based on mitochondrial *cytB*, *ND2*, and *16S rDNA *sequences. Maximum likelihood analysis, values at nodes are for bootstrap percentages from 500 replicates, only nodes supported by 70% or greater are shown. * by species name designates the organisms for which the complete mitochondrial sequence has been determined.

Table 1 gives the properties and the accession numbers of the mitochondrial DNA sequences determined in this study. An unrooted phylogenetic tree showing the relationships of whiteflies, psyllids and aphids, based on mitochondrial *cytB *(partial), *ND1*, and *16S rDNA *is presented in Fig. 1. A similar tree is obtained when the amino acid sequence of CytB (partial) and ND1 is used. The sole difference is the position of *Neomaskellia andropogonis *which becomes part of the cluster containing *Bemisia tabaci*, *Tetraleurodes acaciae*, *Aleurochiton aceris*, and *Trialeurodes vaporariorum*. Whiteflies, psyllids and aphids have associations with different primary endosymbionts that are transmitted vertically and are essential for the survival of the insect host [2-6]. The time for the establishment of these endosymbiotic associations and the emergence of the composite organism is generally estimated to be between 100 and 200 million years ago [2]. The representative species chosen for study (Fig. 1) probably span the range of diversity within whiteflies, psyllids, and aphids. The maximum % difference in the DNA sequence of these organisms is 33.5 % for whiteflies, 29.7% for psyllids and 13.1% for aphids suggesting that the rate of mitochondrial sequence change in aphids is considerably less than that in whiteflies and psyllids. Resolution of the order of branching among these insect groups is not possible using mitochondrial sequences, since due to their rapid rate of change they are saturated.

**Table 1:** Properties and accession numbers of mitochondrial DNA sequences determined in this study.

Organism	Type	Mitochondrial sequence	Size (bp)	G+C content	GenBank accession number
*Bemisia tabaci*	whitefly-A	complete	15,322	25.9	AY521259
*Tetraleurodes acaciae*	whitefly-B	complete	15,080	28.0	AY521626
*Neomaskellia andropogonis*	whitefly-C	complete	14,496	18.7	AY572539
*Aleurochiton aceris*	whitefly-D	complete	15,388	22.1	AY572538
*Trialeurodes vaporariorum*	whitefly-Y	complete	18,414	27.7	AY521265
*Aleurodicus dugesii*	whitefly-Y	complete	15,723	13.8	AY521251
*Pachypsylla venusta*	psyllid	complete	14,711	26.3	AY278317
*Schizaphis graminum*	aphid	complete	15,721	16.1	AY531391
*Bemisia argentifolii*	whitefly-A	*cytB-COIII*	4, 796	23.2	AY521257
*Bemisia *sp.	whitefly-A	*12S-COIII*	985	19.4	AY572845
		*cytB-12S*			AY521257^a^
*Aleuroplatus *sp.	whitefly-B	*cytB-COIII*	4, 540	27.4	AY521256
*Tetraleurodes mori*	whitefly-B	*cytB-COIII*	4,416	25.3	AY521263
*Vasdavidius concursus*	whitefly-C	*cytB-COIII*	3,374	20.0	AY648941
*Siphonius phillyreae*	whitefly-D	*cytB-12S*	4,561	22.3	AY521268
*Bactericera cockerelli*	psyllid	*cytB-12S*	3,077	28.0	AY601890
*Calophya schini*	psyllid	*cytB-12S*	3,044	26.3	AY601891
*Glycaspis brimblecombei*	psyllid	cytB-12S	3,081	26.8	AY601889
*Diuraphis noxia*	aphid	*cytB-12S*	3,180	15.8	AY601892
*Melaphis rhois*	aphid	*cytB-12S*	3,184	17.0	AY601894
*Schlechtendalia chinensis*	aphid	*cytB-12S*	3,188	16.1	AY601893
*Daktulosphaira vitifoliae*	phylloxera	*cytB-12S*	3,215	22.6	AY601895

^a^From [6].

### Mitochondrial genomes with a similar gene order

**Figure 2:**
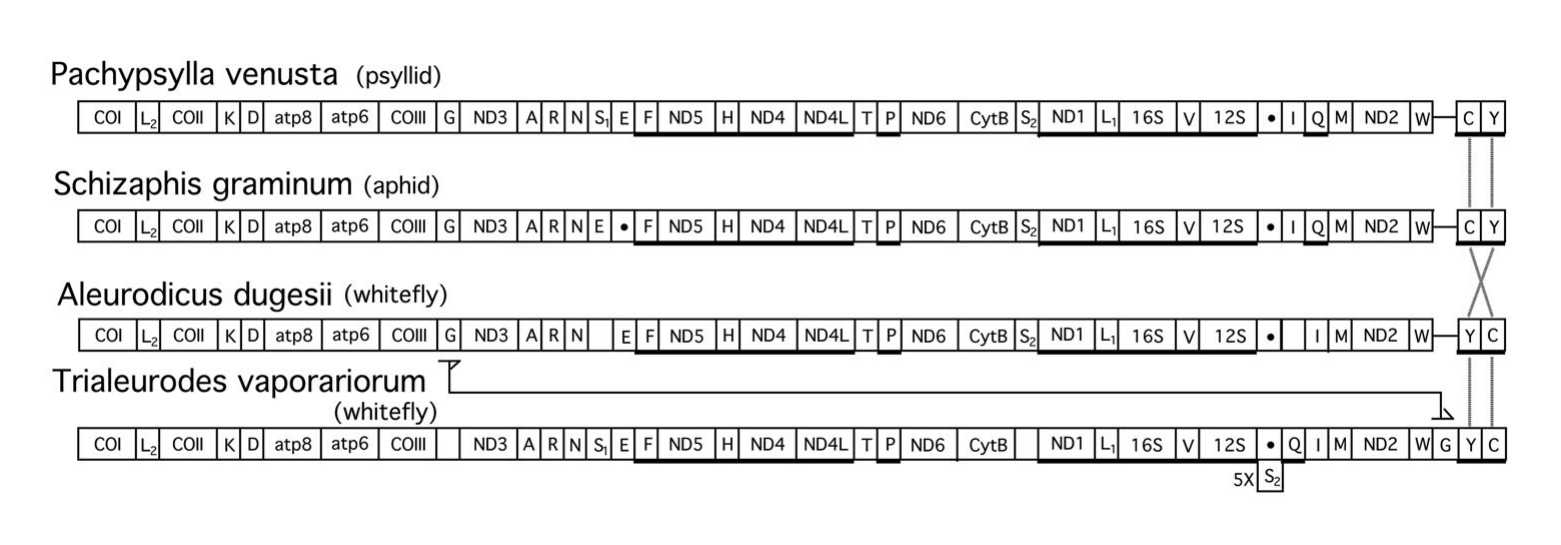
**Mitochondrial gene arrangements of psyllids, aphids, and selected whitefly species all of which have a highly similar gene order. **Genes are transcribed from left to right except for the underlined genes which are transcribed in the opposite direction. Dot in box indicates a putative origin of replication and/or a region of direct repeats. Empty box indicates 40–60 nt that do not code for a readily identifiable tRNA. Horizontal bar between genes indicates that they are contiguous with little or no nucleotides between them. The change in the position of *tRNA-G *in *T. vaporariorum *is traced by lines and the 5X preceding the box containing *tRNA-S2*, indicates that this putative tRNA is present five time in a direct repeat.

**Figure 3:**
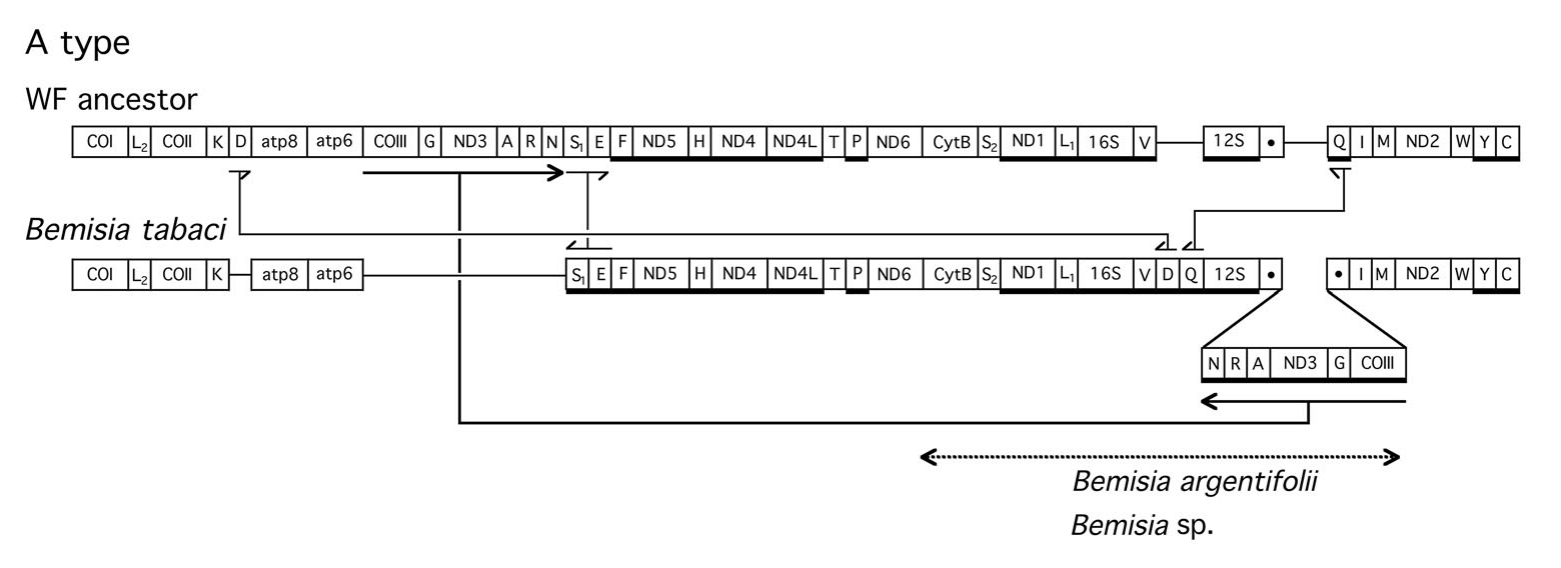
**Differences of the A type gene order from the postulated whitefly ancestral gene order. **The principal changes are indicated by thick lineswith complete arrowheads. Direction of the arrowheads or half arrowheads indicates direction of transcription. Figure legend same as for Fig. 2. Dashed double headed arrow indicates the sequenced genes from other listed whitefly species.

**Figure 4:**
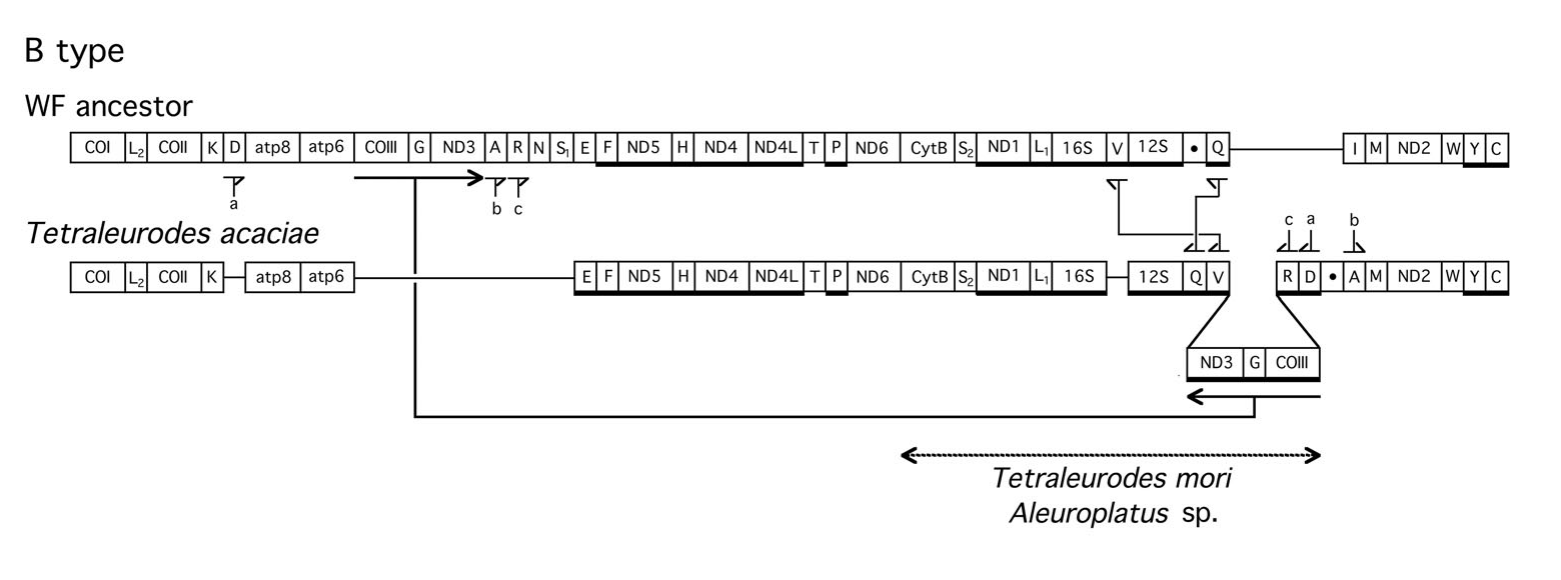
**Differences of the B type gene order from the postulated whitefly ancestral gene order. **Figure legend same as in Fig. 2 and 3.

**Figure 6:**
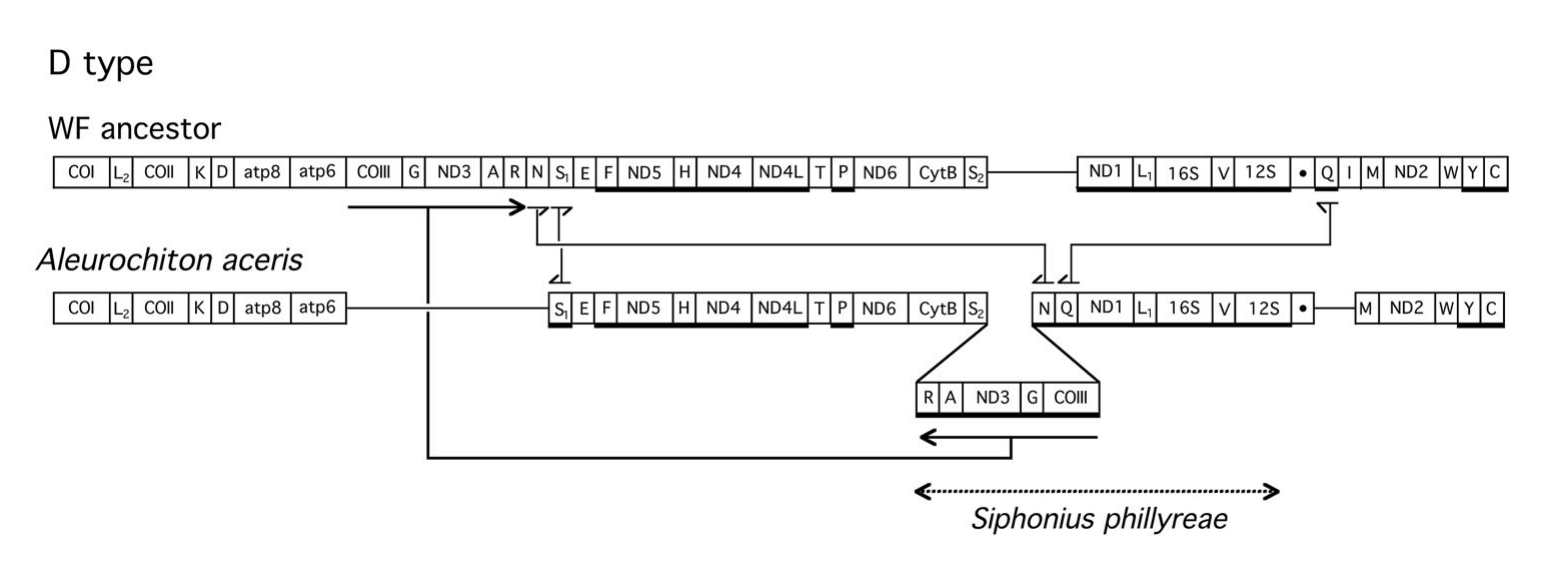
**Differences of the D type gene order from the postulated whitefly ancestral gene order. **Figure legend same as in Fig. 2 and 3.

With some notable exceptions, within the class Insecta the order of the mitochondrial genes is highly conserved and has led to the proposal of an ancestral gene order [10,18]. An identical or a similar gene order has been observed in the mitochondrion of *Pachypsylla venusta *(psyllid), *Schizaphis graminum *(aphid), as well as *Aleurodicus dugesii *and *Trialeurodes vaporariorum *(whiteflies) (Fig. 2). In pysllids and aphids, *tRNA-C *is followed by *tRNA-Y *(Fig. 2, extreme right) which corresponds to the ancestral Insecta gene order. In most whiteflies (Fig. 2, 3, 4, 6), the order of these tRNA genes is reversed and this probably constitutes the whitelfly ancestral gene order. In the mitochondria of *T. vaporariorum*, *tRNA-G *is transposed from its position between *COIII *and *ND3 *to a position between *tRNA-W *and *tRNA-Y *(Fig. 1). *tRNA-S1 *was not detected in the mitochondria of *S. graminum*; this tRNA and *tRNA-Q *was not detected in *A. dugesii*.

### Mitochondria of whiteflies with transposition of *COIII-(tRNA-G)-ND3-(tRNAs-A-R-N)*

**Figure 5:**
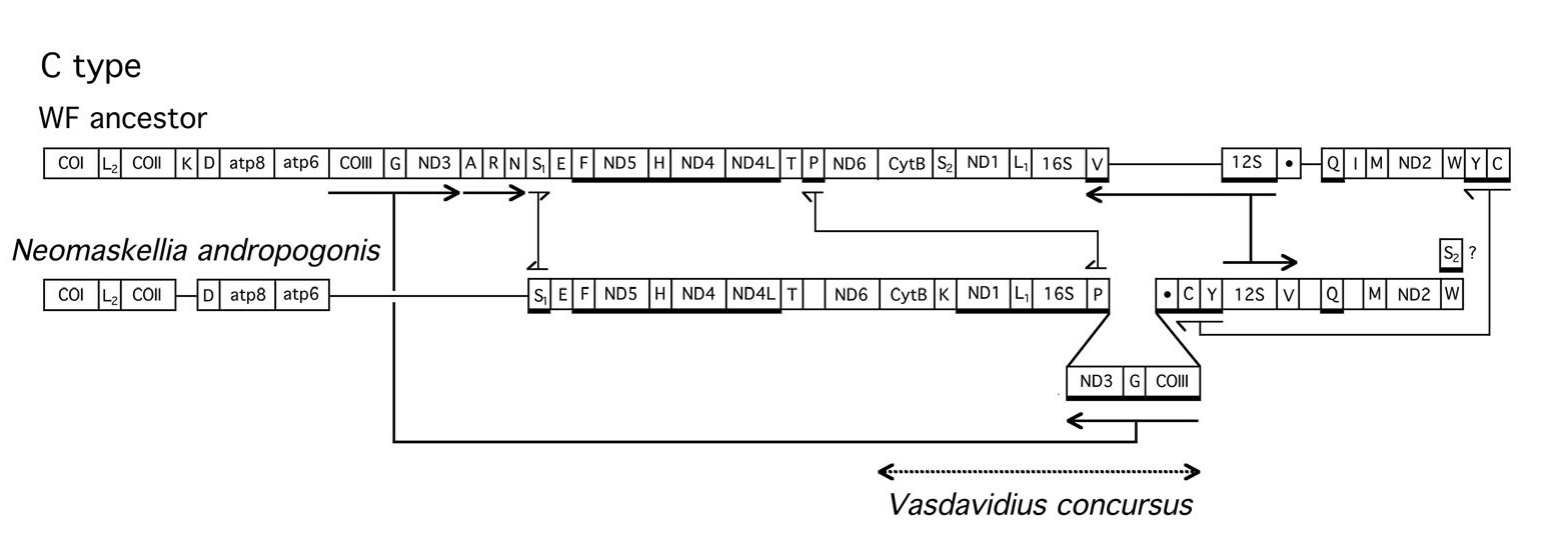
**Differences of the C type gene order from the postulated whitefly ancestral gene order. **Figure legend same as in Fig. 2 and 3.

A number of whitefly mitochondria had transpositions of DNA fragments containing *COIII-(tRNA-G)-ND3-(tRNAs-A-R-N)*. In most cases in which these genes are removed, there is a change in the direction of transcription of the adjacent downstream *tRNA-S1 *from clockwise to counter clockwise (Fig. 3, 5, 6). There is variation in the mitochondrial position into which these genes are transposed. In addition, there are differences with respect to the retention of the number and the order of the excised tRNA genes at the mitochondrial location in which the genes are inserted. The maximal insertion involves all of the genes from the excised fragment in their original order (Fig. 3) *(tRNAs-A-R-N)-ND3-(tRNA-G)-COIII)*, the minimal insertion involves *ND3-(tRNA-G)-COIII *(Fig. 4, 5). In all insertions, the transcription direction is altered from that in the original position. Based on the location of the insertions and the adjacent genes, we have subdivided these transpositions into four types (A-D) (Fig. 3,4,5,6). In all cases, it would appear that the excision involved the removal of *COIII-(tRNA-G)-ND3-(tRNAs-A-R-N)*. However, the DNA that is inserted always contains *COIII-(tRNA-G)-ND3 *and may contain all or only some of the tRNA genes.

Transposition of the A type is shown in Fig. 3. In this case, *COIII-(tRNA-G)-ND3-(tRNAs-A-R-N) *is removed from the inferred ancestral position and placed between *12S rDNA *and *tRNA-I*. Additional changes involve the position of *tRNA-D*, *tRNA-Q *and the direction of transcription of *tRNA-S1 *and *tRNA-E*. There is a total of 5 difference between the A type gene arrangement and the ancestral whitefly gene order. Sequence determination of smaller DNA fragments from two related species (*cytB-COIII*) were consistent with the same gene order (Fig. 3).

Transposition of the B type is shown in Fig. 4. In this case, *ND3-(tRNA-G)-COIII *is inserted into a location downstream of *12S rDNA *and is bounded by tRNAs that have also changed locations (*tRNAs-Q-V *and *tRNAs-R-D*). In addition, the position of *tRNA-A *is changed as compared to the ancestral position. There were 6 differences from the putative ancestral gene order. No tRNA genes for N, S1, and I were detected. Sequence determination of a smaller DNA fragment (*cytB-COIII*) from two related species was consistent with the same gene order.

Transposition of the C type is shown in Fig. 5. In this case, *ND3-(tRNA-G)-COIII *is inserted downstream of *16S rDNA *between *tRNA-P *and *tRNA-C*. Another major difference is the change in the direction of the transcription of *tRNA-V *and *12S rDNA*. Other differences include the change in the order and the position of *tRNA-Y *and *tRNA-C*, the change of position of *tRNA-P*, and the direction of transcription of *tRNA-S1*. Putative *tRNA-W *is transcribed clockwise. A small change in the span of the DNA fragment resulted in a putative *tRNA-S2*, transcribed counter clockwise. The initially adjacent *tRNAs-A-R-N *as well as *tRNA-I *were not detected. There was a total of 7 differences between the whitefly ancestral gene order and the C type gene order. Sequence determination of a fragment of mitochondrial DNA from a related whitefly species was consistent with the C type gene order.

The D type gene order is shown in Fig. 6. In this case,*(tRNAs-R-A)-ND3-(tRNA-G)-COIII *is found after *tRNA-S2 *and before *tRNA-N*. Additional differences from the ancestral gene order involve the change in position of *tRNA-N *and *tRNA-Q *and the direction of transcription of *tRNA-S1. tRNA-I *was not detected. The total number of differences between the ancestral gene order and the D type gene order is 4. The sequence of a mitochondrial DNA fragment from a related species indicated a gene order of the D type (Fig. 6).

### PCR-based screening for excision of *COIII-(tRNA-G)-ND3-(tRNAs-A-R-N) *and identification of transposition types

**Figure 7:**
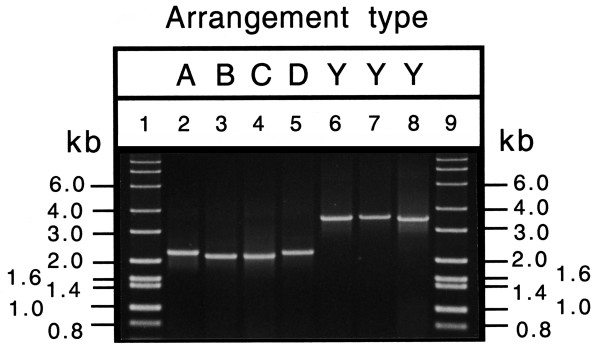
**Agarose gel electrophoresis of PCR products amplified from whole insect DNA using primers complementary to regions encoding *COII *and *ND5***. A, B, C, D, refers to different gene arrangement types; Y, ancestral arrangement. Lanes 1 and 9 molecular size markers; lane 2, *Bemisia tabaci*; lane 3, *Tetraleurodes acaciae*; lane 4, *Neomaskellia andropogonis*; lane 5, *Aleurochiton aceris*; lane 6, *Aleyrodes elevatus*; lane 7, *Trialeurodes vaporariorum*; and lane 8, *Aleurodicus dugesii*.

We have devised a set of oligonucleotide primers complementary to *COII *and *ND5 *that allow the amplification of the DNA between these two genes. The size of the resulting fragments is a potential indication of the presence or absence of *COIII-(tRNA-G)-ND3-(tRNAs-A-R-N) *between *COII *and *ND5 *(Fig. 2). Fig. 7 shows the results obtained with insects containing mitochondria that have these genes in the ancestral position (lanes 6–8, bands of 3.7 kb) and those in which they have been excised from this position (lane 2–5, bands of 2.2 to 2.3 kb).

**Figure 8:**
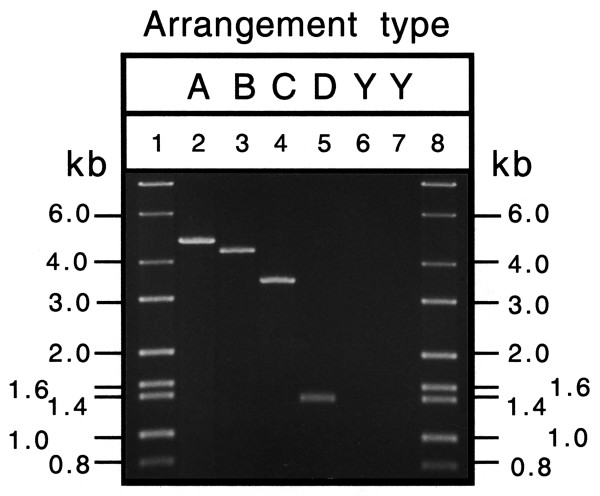
**Agarose gel electrophoresis of PCR products amplified from whole insect DNA using primers complementary to regions encoding *COIII *and *cytB*. **A, B, C, D, refers to different gene arrangement types; Y, ancestral arrangement. Lanes 1 and 8 molecular size markers; lane 2, *Bemisia tabaci*; lane 3, *Tetraleurodes acaciae*; lane 4, *Neomaskellia andropogonis*; lane 5, *Aleurochiton aceris*; lane 6, *Trialeurodes vaporariorum*; and lane 7, *Aleurodicus dugesii*.

In addition, we have devised a set of PCR primers that allow the distinction of the four types of transpositions. Using oligonucleotide primers complementary to *COIII *and *cytB*, the PCR fragments shown in Fig. 8 were obtained. The sizes characteristic of arrangement types A, B, C and D, were 4.9, 4.5, 3.5, and 1.5 kb, respectively.

### Non-coding regions

**Figure 9:**
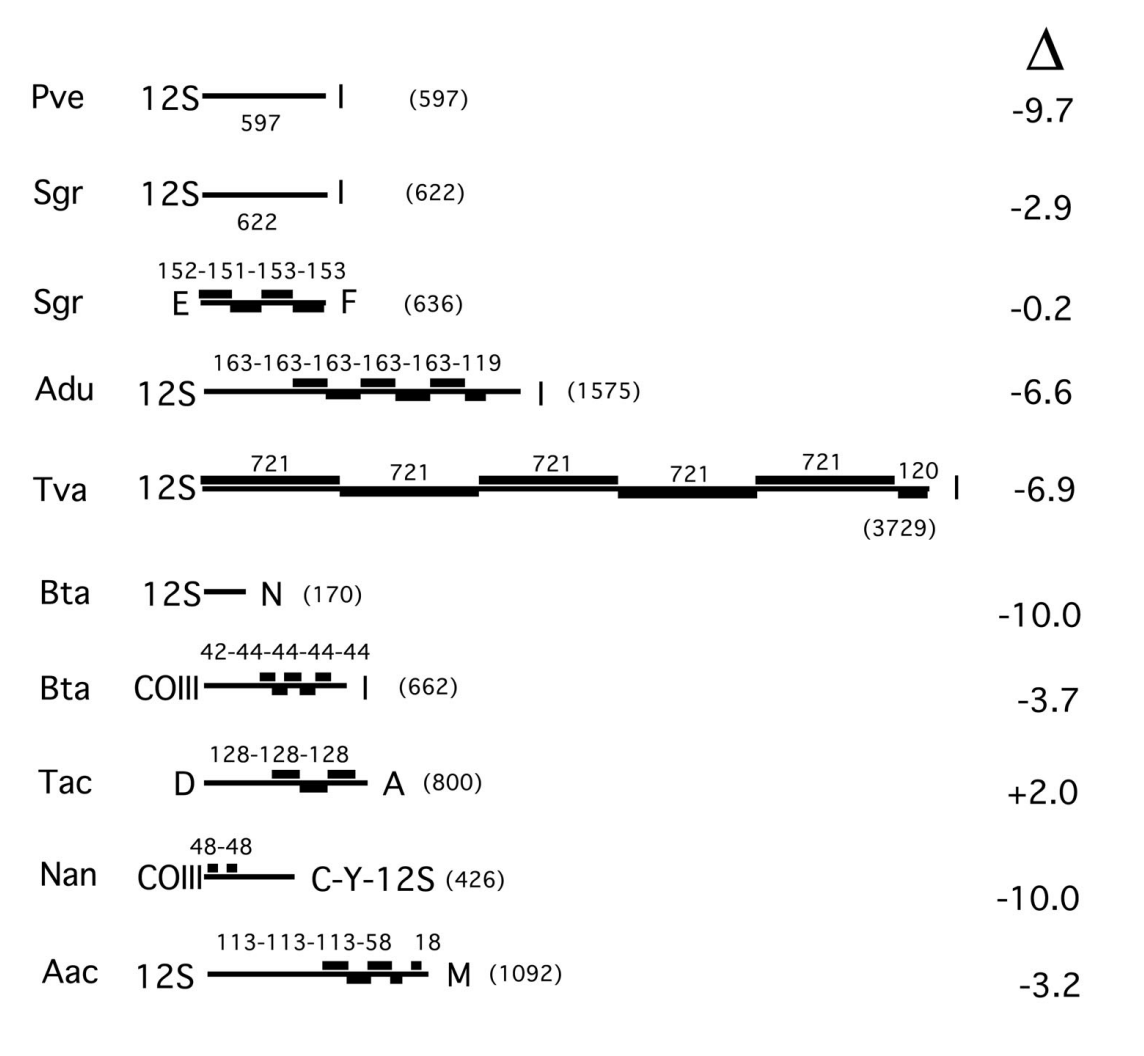
**Summary of the properties of non-coding regions found in the mitochondrial genomes of psyllids, aphids and whiteflies. **Letter abbreviations for amino acids, denote tRNAs for the designated amino acid; thin lines, length of non-coding region; thick lines, direct repeats; numerals above line, length of direct repeats, numbers in parentheses at end of fragment denote its length in bp; column of numbers at right represent the difference between the G+C content of the full mitochondrial genome and the considered segment. Pve, psyllid, *Pachypsylla venusta*; Sgr, aphid, *Schizaphis graminum*; Adu, whitefly, *Aleurodicus dugesii*; Tva, whitefly, *Trialeurodes vaporariorum*; Bta, whitefly, *Bemisia tabaci*;Tac, whitefly, *Tetraleurodes acaciae*; Nan, whitefly, *Neomaskellia andropogonis*; *Aac*, *whitefly*, *Aleurochiton aceris*.

Mitochondrial genomes of insects are very compact. The principal non-coding segments of the genome are a low G+C content region usually following *12S rDNA *[10-12]. The low G+C region usually has stretches of "T"s or "A"s as well as multiples of the sequence "TA." Another feature of this region may be inverted and direct repeats. Fig. 9 presents a diagrammatic summary illustrating some of the properties of the non-coding regions of the mitochondria of the studied insects. Only direct repeats and their sizes are indicated in this figure. No consistent pattern of inverted repeats was found and these are not indicated in the diagrams. All of the non-coding regions in the vicinity of *12S rDNA *had a G+C content lower than the G+C content of the full genome (Fig. 9). The decrease ranged from 3.2 to 10.0%. Some of these regions of lower G+C content, adjacent to *12S rDNA*, contained direct repeats (Fig. 7, Adu, Tva, Aac). In Bta and Nan (Fig. 9), the direct repeats were in a non-coding region following *COIII *that also had a decrease in the G+C content. The noncoding regions of Tac, containing direct repeats, had a G+C content that was actually higher than that of the full Tac mitochondrial genome. However, the segment before the repeats had regions with a lower G+C content. In Sgr (Fig. 9), the direct repeats were between *tRNA-E *and *tRNA-F *and had essentially no decrease in the G+C content. In this organism and Pve (Fig. 9), the region following *12S rDNA *had a decrease G+C content but did not contain substantial direct repeats.

### tRNA anticodons

In general, the anticodons found in the tRNAs of the mitochondria of whiteflies, psyllids, and aphids were those expected of insect mitochondria [12]. Some exceptions were "TTT" (instead of CTT) for *tRNA-K *for *B. tabaci *and *A. dugesii*, and "TCT"(instead of GCT) for *tRNA-S1 *for *B. tabaci*, *A. aceris*, *N. andropogonis *and *T. vaporariorum*. The latter codon maybe the usual *tRNA-S1 *anticodon in whiteflies; this tRNA was not detected in *T. acaciae *and *A. dugesii*.

## Discussion

**Figure 10:**
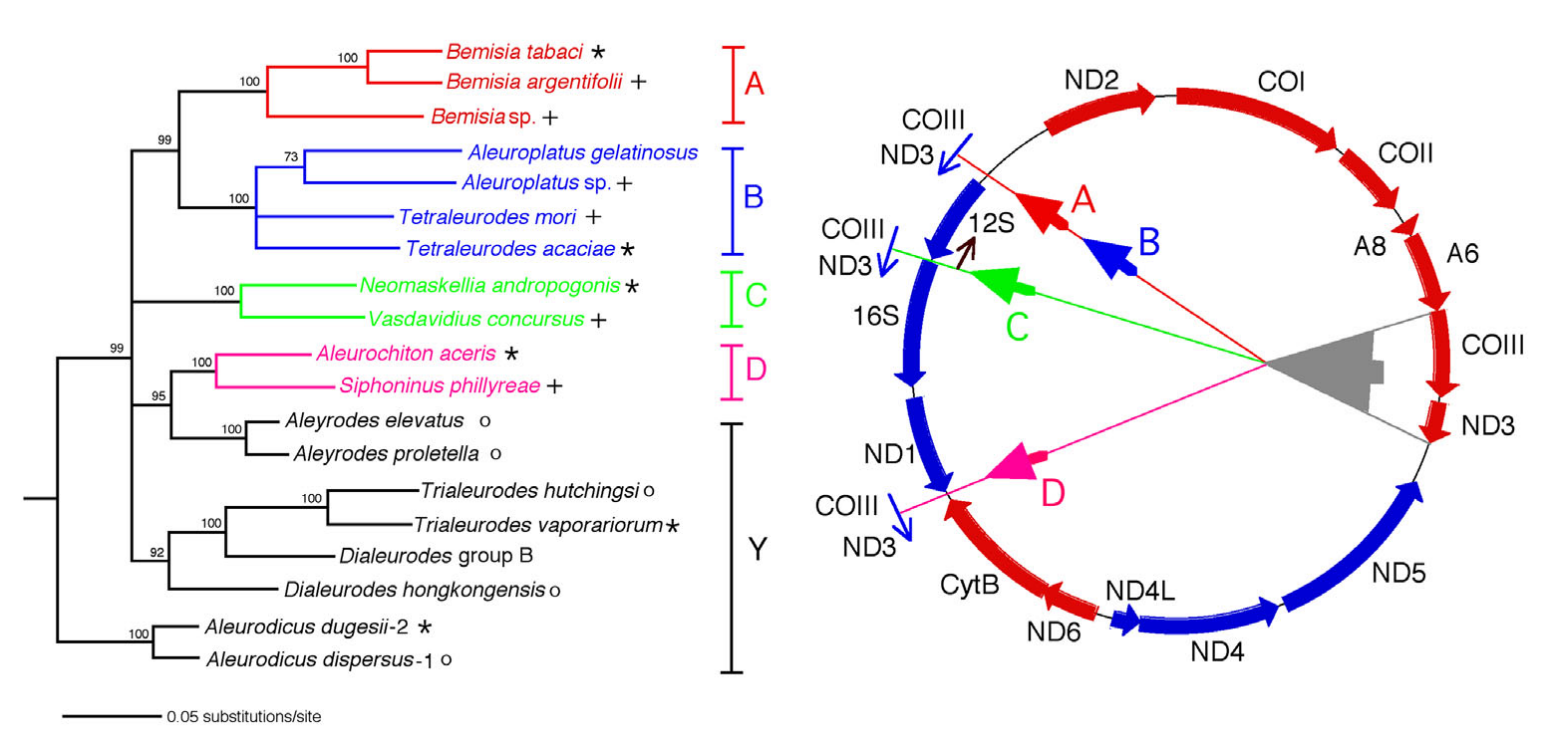
**Summary of the major transpositions occurring in the mitochondria of whiteflies and the relationship of these changes to the phylogeny of whitefly species**. The phylogenetic tree was obtained on the basis of combined mitochondrial *cytB-N22-16S rDNA *and *Portiera *endosymbiont 16S* and 23S* rDNA using the maximum likelihood method. Numbers at nodes correspond to bootstrap values after 500 replicates. The combination of the host and endosymbiont sequence data is justified by their cospeciation [6]. A, B, C, D indicate transposition type; Y, indicates mitochondria with an ancestral gene arrangement. Large arrowhead in mitochondrial genome indicates the original position of the transposed genes. Small arrowheads indicate the position of the insertion of the genes. Arrows outside circle indicate the direction of the transcription of the transposed genes. Arrow by the arrowhead of the B type transposition indicates the changed direction of transcription of the *12S rDNA*. tRNAs have been omitted. (*), by species names indicate that the full mitochondrial genome was sequenced. (+), by species names indicates that a DNA fragment containing all or a part of the gene encoding for *COIII *and adjacent genes was sequenced. (o), by species name indicates that using oligonucleotide primers to *COII *and *ND5 *a PCR product was obtained corresponding to a size that was consistent with the presence of *COIII-(tRNA-G)-ND3-(tRNAs-A-R-N) *in the ancestral position (Fig. 8).

The novel aspect of this study is the finding that whitefly mitochondria contain a region of their genome spanning *COIII-(tRNA-G)-ND3-(tRNAs-A-R-N) *that is prone to excision followed by insertion as a unit or as fragments in different parts of the mitochondrial genome. Based on the collection of whiteflies we have examined, this event occurred three or four different times in the ancestors of the studied species. These conclusions are summarized in Fig. 10 where the phylogeny of the whiteflies is compared to the gene arrangement types. The designation Y refers to the ancestral arrangement established in the species under this designation. Species bracketed under A and B have a similar insertion position for *COIII-(tRNA-G)-ND3*) (Fig. 3, 4) but differ in adjacent tRNAs, so that it is possible these differences followed the insertion of the transposed fragment in a common ancestor. The positions of the transpositions in species of clusters C and D are very different and are probably the results of independent events. For purposes of this discussion we have chosen the simplest interpretation but this does not exclude other more complex scenarios. Cluster C is of additional interest since it is related to two species of *Aleyrodes *that have the ancestral (Y) arrangement. From the 16S*-23S* rDNA sequence divergence of *Portiera *(the primary endosymbiont of whiteflies) and the estimated rate of endosymbiont sequence change [24], it is possible to estimate the time of divergence of cluster C and the two *Aleyrodes *species. This value corresponds to 30–60 million years ago which is the maximum time for the occurrence of the transposition in an ancestor of cluster C.

The excision of the same mitochondrial fragment at least four times during the evolutionary history of whiteflies suggests that this fragment is prone to transposition. In spite of the apparent similarity of the excisions, we have not been able to find any conserved sequence properties either adjacent to the region of the excised fragment or adjacent to its insertion site. The excision appears to be associated with a change in the direction of transcription of the previously adjacent *tRNA-S1 *(Fig. 3, 5, 6) and a change in the direction of transcription of the relocated fragments. As previously noted the order of the mitochondrial genes is conserved in most insects [10,14]. The major exceptions are within the three hemipteroid orders Phthiraptera, Psocoptera, and Thysanoptera [18,20,21]. The rearrangements are different within these three orders, being rather extreme in the Thysanoptera. In most insects, the order of the rRNA genes is *16S-(tRNA-V)-12S *and the genes are transcribed in the counterclockwise direction [10,18]. A major exception is in the mitochondrion of *Thrips imagines *where these two genes are distant from each other and transcribed in opposite orientations [18]. In the C type gene order, there is an inversion of *12S-(tRNA-V) *that is possibly associated with the insertion of *ND3-(tRNA-G)-COIII *between *16S *and *12S rDNA *(Fig. 5, 10). This situation resembles that found in *Thrips imagines *in that the rRNA genes are transcribed in opposite directions. In whiteflies, besides rearrangements involving *COIII-(tRNA-G)-ND3-(tRNAs-A-R-N)*, there are also substantial rearrangements involving single tRNAs. The physiological significance (if any) of these rearrangements is not known. Genes that are highly expressed (*16S*, *12S rDNA*) when separated and transcribed in opposite orientations would have to become part of different transcription units. In addition, we are not certain of the validity or significance of our inability to find a few of the tRNAs. In some cases, this may stem from our inability to recognize them. In other cases, such as *tRNAs-A-R-N *that are absent in the type C gene order, there would not appear to be any room for these genes on the mitochondrion and it might be that the tRNAs for these amino acids are provided by the host [25].

The mitochondrion of *A. dugesii *has a G+C content of 13.8 moles % (Table 1). All the other sequenced whitefly mitochondria have G+C contents of 18.7 to 27.7 moles % (Table 1). On the basis of morphological classification, *Aleurodicus *has been placed into a subfamily Aleurodicinae, while the remaining whitefly species listed in Fig. 10 have been placed into the subfamily Aleyrodinae [1]. This separation is supported by a phylogenetic analysis of mitochondrial DNA, host 18S rDNA, as well as *Portiera *DNA from different whitefly species [6-8]. It is possible that the common ancestor of whiteflies had a higher G+C content in its mitochondria and that in *Aleurodicus *there was a decrease. Alternatively, it is possible that the ancestral G+C content was low and increased in the Aleyrodinae.

Our work points to the uncertainty inherent in making generalizations from one or a few organisms assumed to be representative of a group. We were fortunate that in the whiteflies the first mitochondrion we chose to study was that of *B. tabaci *which had an altered gene order. Had we started with our second or third choice (*T. vaporariorum*, *A. dugesii*) we would have concluded that the whiteflies have the ancestral mitochondrial gene order and not pursued further studies of mitochondria within this group of insects. Previously, evidence was found of a correlation between the rate of nucleotide sequence change and the rate of gene rearrangement [26]. If this has general applicability one would expect conservation of the mitochondrial gene order in aphids which have a low rate of sequence change (Fig. 1) and perhaps some changes in the gene order of psyllids as has been observed with whiteflies. The relatively localized different changes observed in several whitefly lineages may be of use in the study of the phylogeny and taxonomy of these organisms as is already indicated from the relatively small sample of organism studied in the present work.

## Conclusions

Psyllids, aphids, and many whiteflies have mitochondria in which the order of the genes resembles the proposed Insecta ancestral gene order. However, in a variety of whitefly species there is a change in the gene order. In these organisms, there is an excision of a DNA segment containing *COIII-(tRNA-G)-ND3-(tRNAs-A-R-N) *from the ancestral position, between *atp6 *and *tRNA-S1*, and the insertion of all of these genes or fragments containing *COIII-(tRNA-G)-ND *and tRNAs into different locations on the mitochondrial genome. On the basis of the insertion positions, four gene arrangement types were identified. A phylogenetic analysis of 19 whitefly species involving mitochondrial and endosymbiont genes showed that each arrangement type was characteristic of a cluster of related whitefly species indicating that the transposition occurred in a common ancestor of the related species. The reason for the "restlessness" of this DNA segment in whiteflies and the physiological significance of these rearrangements are not known.

## Methods

### Amplification and sequencing of mitochondrial genomes

In all cases, the starting material was whole insect DNA that was prepared and used in a previous study [6]. In our initial attempts at cloning mitochondrial DNA, we used methods previously developed for obtaining clones of insect endosymbiont DNA that have been described in detail [27]. In outline this involved obtaining a homologous probe for *COI *using previously described primers [28], followed by restriction enzyme and Southern blot analysis of insect DNA. Appropriate sized fragments were electroeluted from agarose gels and cloned into λ-ZAP (Stratagene, La Jolla, California). Following excision of the insert-containing plasmid, the DNA sequence was determined using a double stranded nested deletion kit (Pharmacia, Piscatawy, New Jersey) and where necessary custom-made oligonucleotides. As new sequence data was acquired for the mitochondria of several insect species our ability to design more specific oligonucleotide primers was improved. This allowed us to use pairs of primers, in combination with PCR, to obtain the full mitochondrial genome in 2–4 overlapping fragments. Conserved regions of the whitefly mitochondrial genome that are of use for the design of oligonucleotide primers, based on comparisons of six mitochondrial genomes, are given in Table 2. Usually the oligonucleotide primers had added sequences at the 5'-ends for restriction enzymes.

**Table 2:** Oligonucleotide primers for PCR amplification of whitefly mitochondrial DNA fragments.^a^

Primer	Gene	Position on AY5212656	Nucleotide sequence of conserved regions (5'->3')
F-COI-1	COI	172–221	TCWCATGCWT TTATYATAAT TTTTTTYATR ACWATGCCTT TDGTWATTGG
F-COI-2	COI	673–710	GAYCCHATTT TRTATCAACA YTTDTTTTGATTTTTTGG
R-COI	COI	1133–1069	ACATAATGAA AATGDGCAAC AACAAAATAWGTATCATGHA RACAHACATC HACHGAAGAA TTACC
F-COII	COII	1845–1876	CCTTCTATYC GDATTTTDTA TYTAATRGAT GA
R-COII	COII	2093–2067	AGGAACHGTY CAAGAATGHA AAACATC
F-COIII	COIII	3854–3879	TTAACWGGHT TTCAYGGNTT HCATGT
R-COIII	COIII	4002–3974	CARACWAHRT CDACRAAATG TCAGTATCA
F-CYTB	CYTB	9169–9215	GCTTTTATRG GBTATATYTT RCCTTGRGGY CARATATCTT TTTGRGG
R-CYTB	CYTB	9566–9544	GCTATAATAA AATTTTCTGA ATC
F-ND1	ND1	10275–10314	ATTCAATRTT AAAWCCWGAA ATWARYTCTG AYTCTCCTTC
R-ND1	ND1	10506–10484	CAAYTAATTT CDTATGAAAT TAA
F-16S-1	16S	11053–11087	ACCTGGCTTA CGCCGGTCTG AACTCAGATC ATGTA
F-16S-2	16S	11202–11220	GCTGTTATCC CTTAGGTAA
R-16S-1	16S	11377–11354	AAAAGACAAR AAGACCCTTT AGAA
R-16S-2	16S	11526–11483	TTAAATAGCT GCAGTAWATT DACTGTACTA AGGTAGCATA ATAA
F-12S-1	12S	12273–12308	ACTTTCCAGT AADTTTACTT TGTTACGACT TATCTT
F-12S-2	12S	12318–12342	AAGAGTGACG GGCRATTTGT ACATA
R-12S-1	12S	12643–12619	CTTCAAACTT AAAAAATTTG GCGGT
R-12S-2	12S	12861–12843	GTGCCAGCAG TWGCGGTTA

^a^Underlined sequences indicate primers used in this study. ^b^Mitochondrial genome of *Trialeurodes vaporariorum*.

We will illustrate the approach by describing how the full genome of the mitochondrion of *T. acaciae *was obtained in three overlapping fragments. Using primers F-CYTB and R-12S-2 (Table 2) and PCR, a 3.6 kb DNA fragment was obtained. Similarly, using the pairs of primers F-COI-2 and R-CYTB and F-12S-2 and R-COI fragments of 7.3 and 5.5 kb, respectively, were obtained. For fragments of 4 kb or less, the PCR reaction mixture (10 ul) contained 10 ng insect DNA, 1 ug bovine serum albumin, 5 mM MgCl_2_, 0.2 mM dNTP, 10 pmoles of each primer, 0.6 U Bio-X-Act DNA polymerase, in Opti-Buffer (Bioline, London, United Kingdom). The PCR program was 94°C for 3 min, 30 cycles of, 94°C 30 sec, 55.0–65.0°C (predetermined optimal annealing temperatures) 30 sec, 70° 5 min, followed by 70°C 10 min. For the 5.5 and 7.3 kb DNA fragments, the PCR reaction mixture was modified by the increase of dNTPs to 0.3–0.4 mM and Bio-X-Act to 0.8 U. The PCR program was 94°C for 2 min, 10 cycles of 92°C 20 sec, 55.0–65.0°C (predetermined optimal annealing temperatures) 30 sec, 68° 10 min, followed by 20 cycles of 92°C 20 sec, optimal annealing temperature 30 sec, 68°C 10 min with increases of 15 sec each cycle, followed by 68°C 10 min. The DNA fragments were purified by means of the Wizard SV gel and PCR clean-up system (Promega, Madison, Wisconsin) as directed by the manufacturer. Following digestion with restriction enzymes the mitochondrial DNA fragments were cloned into pBluescript (Stratagene). In some cases where difficulty was experienced with using this vector due to possible toxicity of the inserts, the low copy number plasmid pWSK130 was used [29]. The DNA sequence was obtained as described above. Sequences were determined at the University of Arizona (Tucson) LMSE sequencing facility. In some cases, PCR fragments of 1 to 4 kb were directly sequenced after gel purification using custom made oligonucleotide primers.

### PCR amplification of other mitochondrial fragments

*CytB-12S *mitochondrial DNA fragments were amplified and cloned into pBluescript as previously described [6]. *CytB-COIII *DNA fragments were obtained using oligo WF-CYTB-3 (*Bam*HI, *Sac*II; 5'-GCAGGATCCG CGGCCWTGRG GHCAAATATC WTTTTGRGGD GC-3') and WF-COIII-3 (*Kpn*I, 5'-GTGCGGTACC TTCWATTTGR TATTGRCATT TYGTTGA-3') and cloned into pBluescript. *COII-ND5 *DNA fragments (indicative of the presence or absence of COIII-(tRNA-G)-ND3-(tRNAs-A-R-N) were obtained by use of oligo WF-COII (5'-TGYTCAGAAA TYTGTGGRGT TAATCAYAGR TTTATRCC-3') and WF-ND5 (5'-TCAGCMTTAG TYCAYTCWTC AACAYTAGTW ACAGCAGG-3'). *CytB-COIII *fragments (size diagnostic of the arrangement type) were obtained by use of WF-CYTB-1 (5'-TTTATRGGBT ATATYTTRCC TTGRGG-3') and WF-COIII-1 (5'-TATTCWRTWT GATATTGACA TTTYGT-3'). The PCR reaction mixture (10 ul) differed from those above in containing 0.1 mM dNTP, and 0.8 U Bio-X-Act DNA polymerase. The PCR program was 94°C for 5 min, 30 cycles of, 94°C 30 sec, 56.0–63.1°C (predetermined optimal annealing temperatures) 30 sec, 70° 5 min, followed by 70°C 10 min.

### Identification of genes and phylogenetic analyses

The protein-coding and rRNA genes were identified by BLAST searches [30] of GenBank. tRNA genes were identified by tRNAscan-SE [31], DOGMA [32] and in some cases by eye from the anticodons and inferred secondary structures. The methods used for the phylogenetic analyses have been described [6]. In Fig. 1, the phylogenetic analysis of mitochondrial *cytB-ND1-16S *was based on 2730 characters; the analysis in Fig. 10, which besides *cytB-ND1-16S *also included cospeciating endosymbiont 16S*-23S* rDNA [6], was based on 6860 characters.

## List of abbreviations used

### tRNAs

tRNA-one letter amino acid abbreviation (parenthesis three letter amino acid abbreviation followed by anticodons): *tRNA-A *(ala, TGC), *tRNA-C *(cys, GCA), *tRNA-D *(asp, GTC), *tRNA-E *(glu, TTC), *tRNA-F *(phe, GAA), *tRNA-G *(gly, TCC); *tRNA-H *(his, GTG), *tRNA-I *(ile, GAT); *tRNA-K *(lys, TTT or CTT), *tRNA-L1 *(leu, TAG), *tRNA-L2 *(leu, TAA), *tRNA-M *(met, CAT), *tRNA-N *(asn, GTT), *tRNA-P *(pro, TGG), *tRNA-Q *(gln, TTG), *tRNA-R *(arg, TCG), *RNA-S1 *(ser, TCT or GCT), *RNA-S2 *(ser, TGA), *tRNA-T *(thr, TGT), *tRNA-V *(val, TAC), *tRNA-W *(trp, TCA), and *tRNA-Y *(tyr, GTA).

### Other structural genes

*atp6 *(ATP synthase, subunit 6), *atp8 *(ATP synthase, subunit 8), *COI *(cytochrome oxidase, subunit I), *COII *(cytochrome oxidase, subunit II), *COIII *(cytochrome oxidase, subunit III), *ND1 *(NADH dehydrogenase, subunit 1), *ND2 *(NADH dehydrogenase, subunit 2), *ND3 *(NADH dehydrogenase, subunit 3), *ND4 *(NADH dehydrogenase, subunit 4), *ND4L *(NADH dehydrogenase, subunit 4L), *ND5 *(NADH dehydrogenase, subunit 5), *ND6 *(NADH dehydrogenase, subunit 6), *12S *(small subunit of mitochondrial ribosomal DNA [rDNA]), *16S *(large subunit of mitochondrial rDNA), 16S* (small subunit of primary endosymbiont rDNA), 23S* (large subunit of primary endosymbiont rDNA).

### Other abbreviations

%G+C (moles percent guanine+ cytosine in DNA).

## Authors' contributions

MLT cloned and sequenced the mitochondrial genomes of whiteflies. LB cloned and sequenced the mitochondrial genome of a psyllid and an aphid as well as smaller fragments of mitochondrial DNA from psyllids and aphids. PB directed the research and in collaboration with MLT and LB performed the data analysis and wrote the paper.
